# American Medical Association, Section on Stomatology

**Published:** 1902-08

**Authors:** 


### AMERICAN MEDICAL ASSOCIATION, SECTION ON STOMATOLOGY.

### FIRST SESSION.

THE Section on Stomatology met in the upper club-room of the Grand Union Hotel, Saratoga Springs, June 10, 1902, at two P.M., the Chairman, Dr. A. H. Peck, of Chicago, in the chair.

On motion, the programme was accepted as published.

Dr. R. R. Andrews, of Cambridge, Mass., took the chair, and Dr. Peck delivered his address, entitled "Physical Diagnosis as related to Dental College Curriculum."

On motion of Dr. Vida A. Latham, of Chicago, the courtesies of the Section were extended to Professor Gage, of Cornell.

The paper of Dr. Peck was discussed by Drs. J. L. Williams, Vida A. Latham, Professor Gage, Drs. G. V. I. Brown, G. F. Eames, G. Lenox Curtis, M. L. Rhein, E. S. Talbot, D. E. Hoag, E. A. Bogue, and R. R. Andrews, and Dr. A. H. Peck closed the discussion.

Dr. R. R. Andrews of Cambridge read a paper entitled "The Embryology of the Dental Pulp," and gave a lantern-slide exhibition of the embryonal pulp and the dentine tissue.

Dr. Vida A. Latham, of Chicago, read a paper entitled "The Histology of the Pulp," and gave an exhibition of photomicrographs and lantern-slides of developing teeth, illustrating the points of the paper.

The discussion of the papers of Drs. Andrews and Latham was, on motion, postponed to the morning session of June 11.

On motion Dr. J. L. Williams, of Boston, Dr. G. V. I. Brown, of Milwaukee, and Dr. E. A. Bogue, of New York City, were appointed on the Nominating Committee, and the secretary was instructed to cast the ballot.

The Section, on motion, adjourned to Wednesday morning, June 11, at 10 A.M.

SECOND SESSION.

The second session of the Section on Stomatology was called to order by the Chairman at ten A.M. June 11.

The paper on "Notes on the Preparation of Teeth for the Microscope," by Dr. Martha Anderson, Moline, Ill., was read, and was discussed by Drs. Latham, Talbot, Knight, Rhein, Butterfield, and Andrews.

The Nominating Committee reported the names of Dr. M. L. Rhein, of New York City, for chairman, and Dr. Eugene S. Talbot, of Chicago, for secretary. On motion, the chairman cast the ballot, and they were declared elected.

The discussion of the papers of Drs. R. R. Andrews and Vida A. Latham, which had been postponed at the previous session, was taken up and participated in by Drs. Rhein, Bogue, Andrews, Williams, Talbot, Curtis, and Baldwin, Drs. Andrews and Latham closing the discussion.

Dr. Eugene S. Talbot, of Chicago, read a paper entitled "Evolution of the Pulp." Discussed by Dr. Latham, and closed by Dr. Talbot.

Adjourned to two P.M.

THIRD SESSION.

The third session of the Section on Stomatology was called to order at 2.30 P.M. by the Chairman, Dr. A. H. Peck.

A paper entitled "A Comparative Study of the Attachment of Teeth," by Dr. Frederick Noyes, of Chicago, was read, and was discussed by Drs. Talbot and Latham.

Dr. E. A. Bogue, of New York, read a paper entitled "Observations on some Recent Cases of Orthodontia, with Illustrations." The paper was discussed by Drs. Talbot, Latham, and Williams, and Dr. Bogue closed the discussion.

Dr. G. Lenox Curtis, of New York, presented a paper entitled "Electric Ozonation upon Neuralgia." Discussed by Drs. Williams, Bogue, Latham, Eames, and Knight, and Dr. Curtis closed.

Dr. William Knight, of Cincinnati, read a paper entitled "The Modern Dentist from a Medical Stand-Point." The paper was discussed by Drs. Baldwin and Rhein, and closed by Dr. Knight.

Dr. G. F. Eames, of Boston, read a paper entitled "Oral Hygiene." Discussed by Drs. Latham, Talbot, Baldwin, Rhein, and Brown, and closed by Dr. Eames.

On motion, the session adjourned to Thursday, June 12, at two P.M.

FINAL SESSION.

The final session of the Section on Stomatology was called to order at 2.30 P.M. by the chairman, Dr. A. H. Peck.

Dr. Charles Chittenden, of Madison, Wisconsin, read a paper entitled "The Legal Status of the Term 'Reputable,' as applied to Dental Colleges." The paper was discussed by Drs. Brown, Rhein, Baldwin, Talbot, Williams, Peck, and Eames, and Dr. Chittenden closed.

Dr. A. E. Baldwin, of Chicago, offered the following resolution, which was adopted:

*Resolved,* That the Stomatological Section of the American Medical Association heartily endorses the decision of the Wisconsin Supreme Court as to the judicial powers of the State board as to the determination of what constitutes "Reputability;" and we heartily recommend this decision to the National Association of Dental Examiners as a basis for establishing professional reciprocity in our grand country.

Dr. G. V. I. Brown, of Milwaukee, Wis., read a paper entitled "General Nervous Manifestations in Relation to the Jaws and Teeth." The paper was discussed by Drs. Baldwin, Williams, Talbot, Rhein, and Eames, and Dr. Brown closed the discussion.

Adjourned.

[The papers alluded to in the minutes will appear, with discussions in regular order.—ED.]

